# Study design and operational framework for a community-based Malaria Elimination Demonstration Project (MEDP) in 1233 villages of district Mandla, Madhya Pradesh

**DOI:** 10.1186/s12936-020-03458-4

**Published:** 2020-11-16

**Authors:** Harsh Rajvanshi, Praveen K. Bharti, Sekh Nisar, Yashpal Jain, Himanshu Jayswar, Ashok K. Mishra, Ravendra K. Sharma, Kalyan B. Saha, Man Mohan Shukla, Aparup Das, Harpreet Kaur, Suman L. Wattal, Neeru Singh, Altaf A. Lal

**Affiliations:** 1 Malaria Elimination Demonstration Project, Mandla, Madhya Pradesh India; 2 Indian Council of Medical Research, National Institute of Research in Tribal Health (ICMR-NIRTH), Jabalpur, Madhya Pradesh India; 3 Directorate of Health Services, Government of Madhya Pradesh, Bhopal, India; 4 Indian Council of Medical Research, Department of Health Research, Ministry of Health and Family Welfare, New Delhi, India; 5 National Vector Borne Disease Control Programme, Ministry of Health and Family Welfare, New Delhi, India; 6 Foundation for Disease Elimination and Control of India, Mumbai, Maharashtra India; 7 Present Address: Jhpiego, Jaipur, Rajasthan India

**Keywords:** Malaria elimination, Strategic planning, Operational framework, Monitoring and evaluation, India

## Abstract

**Background:**

In the past decade substantial reduction in malaria morbidity and mortality has been observed through well-implemented case management and vector control strategies. India has also achieved a significant reduction in malaria burden in 2018 and has committed to eliminate malaria by 2030. The Mandla Malaria Elimination Demonstration Project (MEDP) was started in 2017 in 1233 villages of District Mandla to demonstrate malaria elimination in a tribal district with hard-to-reach areas was possible using active and passive surveillance, case management, vector control, and targeted information, education and communication campaigns. An operational plan was developed to strengthen the existing surveillance and malaria elimination systems, through fortnightly active case detection to ensure that all cases including those that are introduced into the communities are rapidly identified and treated promptly. The plan also focused on the reduction of human-mosquito contact through the use of Long-Lasting Insecticial Nets (LLINs) and Indoor Residual Spray (IRS). The operational plan was modified in view of the present COVID-19 pandemic by creating systems of assistance for the local administration for COVID-related work while ensuring the operational integrity of malaria elimination efforts.

**Results:**

The use of MEDP study design and operational plan, with its built-in management control systems, has yielded significant (91%) reduction of indigenous cases of malaria during the period from June 2017 to May 2020. The malaria positivity rate was 0.33% in 2017–18, 0.13% in 2018–19, and 0.06% in 2019–20. Mass screening revealed 0.18% malaria positivity in September–October 2018, followed by 0.06% in June 2019, and 0.03% in December 2019, and these were mostly asymptomatic cases in the community. The project has been able to sustain the gains of the past three years during the ongoing COVID-19 pandemic.

**Conclusion:**

This paper provides the study design and the operational plan for malaria elimination in a high-burden district of Central India, which presented difficulties of hard to reach areas, forest malaria, and complex epidemiology of urban and rural malaria. The lessons learned could be used for malaria elimination efforts in rest of the country and other parts of South Asia with comparable demography and epidemiology.

## Background

Malaria has been known to mankind for over 4000 years now. With the Chinese and Indians amongst the first to mention the disease and its symptoms, it was not until 1880 that the malaria parasite was actually demonstrated in a laboratory setting marking the beginning of investigations and the fight against malaria [1]. In the year 2018, there were an estimated 228 million cases worldwide. Fifteen countries in the sub-Saharan Africa and India carried up-to 80% of the global malaria burden.

In the South-East Asia Region (SEAR), progress in malaria control has been noted over the past decade, with the incidence rate falling from 17 cases of the disease per 1,000 population at risk in 2010 to 5 cases in 2018 (a 70% decrease) [2]. Sri Lanka has been malaria free for the past 4 years now and China has reported zero indigenous cases for the past 2 years. Paraguay has been certified as malaria free, and Algeria, Argentina and Uzbekistan have made formal requests for the certification. In 2017, El Salvador reported zero indigenous cases. India has reported a 28% reduction in malaria cases from last year [2], reaffirming that the effectiveness of the existing tools and principles of malaria elimination.

India has a long history of success and struggles with malaria control since the launch of the first formal National Malaria Control Programme (NMCP) in 1953. The tribal areas in India provide the perfect ecology for malaria to thrive with its climatic variations, different vectors, various parasite species, favourable topography and highly susceptible population.

More than 80% of tribal population in India resides in ten states viz*.* Madhya Pradesh, Odisha, Rajasthan, Maharashtra, Jharkhand, Andhra Pradesh, Chhattisgarh, West Bengal, Gujarat and Karnataka. Out of which, Madhya Pradesh and Maharashtra constitute one-fourth of total tribal population. Similarly, more than 80% malaria cases were reported from these tribal states [3].

At the 9th East Asia Summit (EAS) held at Nay Pyi Taw, Myanmar, on November 13, 2013 the leaders of ASEAN member states, including the Republic of India committed to the goal of a malaria-free Asia Pacific by 2030 [4].

Following the Hon’ble Prime Minister’s commitment to make India a malaria free country, the National Vector Borne Disease Control Programme launched National Framework for Malaria Elimination (NFME) in India 2016–2030 and the five year National Strategic Plan (NSP) for Malaria Elimination (2017–2022). The plan provides technical and operational details required to achieve malaria elimination by 2030 [5, 6].

The NSP provides the district level guidance, strategies and roadmap for malaria elimination in a phased manner and its goals are aligned to overall goals of NFME 2016–2030. Complementing the NVBDCP’s efforts to eliminate malaria from India, the Malaria Elimination Demonstration Project’s objective is to demonstrate that malaria elimination is possible in areas with different API in 1233 villages of Mandla district of Madhya Pradesh. The MEDP complimented the routine surveillance, diagnosis and treatment that was provided by the district malaria programme.

Malaria Elimination Demonstration Project (MEDP) is first of its kind Public–Private-Partnership (PPP) between the Indian Council of Medical Research through National Institute of Research in Tribal Health, Govt. of Madhya Pradesh and Foundation of Disease Elimination and Control, which is a CSR initiative of the Sun Pharmaceuticals Industries Limited, with the goal to demonstrate that malaria elimination is feasible with existing tools and within a defined time frame.

Mandla district was chosen because it provided difficulties of hard to reach areas, forest areas, rural and urban areas, and complex epidemiology (both species) and the experience and knowledge of the Indian Council for Medical Research—National Institute for Research in Tribal Health (ICMR-NIRTH) in parasitological, entomological, community-based field investigations, drug treatment, and malaria control and elimination. The experiences of the Indian Malaria Programme were used to develop the MEDP operational plan. The project documents, including IEC materials, training modules, operational tools, and the mobile app (SOCH) are available on the Foundation for Disease Elimination and Control of India (FDEC India) website (www.fdeci.org) and also available to anyone in India and outside India for use.

## Methods

### Study site

Madhya Pradesh is divided into ten administrative divisions. Mandla is a part of the Jabalpur Division and the administrative headquarter of the district. The district has an area of 8771 km^2^, and a recorded population of 1,054,905 [7]. The updated population is 1,140,765 in 2018–2019 according to our household surveillance. The Mandla district has 9 development blocks and 1233 villages. Most of the population is tribal including *Gonds*. The district lies in the Mahakoshal region, and most of the district lies in the basin of the Narmada River.

The Mandla topography also offers a variety of land physical features at different altitudes. The minimum altitude of Mandla in the plains is at 367 to 450 m above the sea level. This altitude rises up-to 901–955 m in the blocks of Mawai and Ghughari with some parts of Bicchiya, all of which have high malaria prevalence along with hard-to-reach areas owing to the mountainous terrain of the areas.

**Fig. 1:**
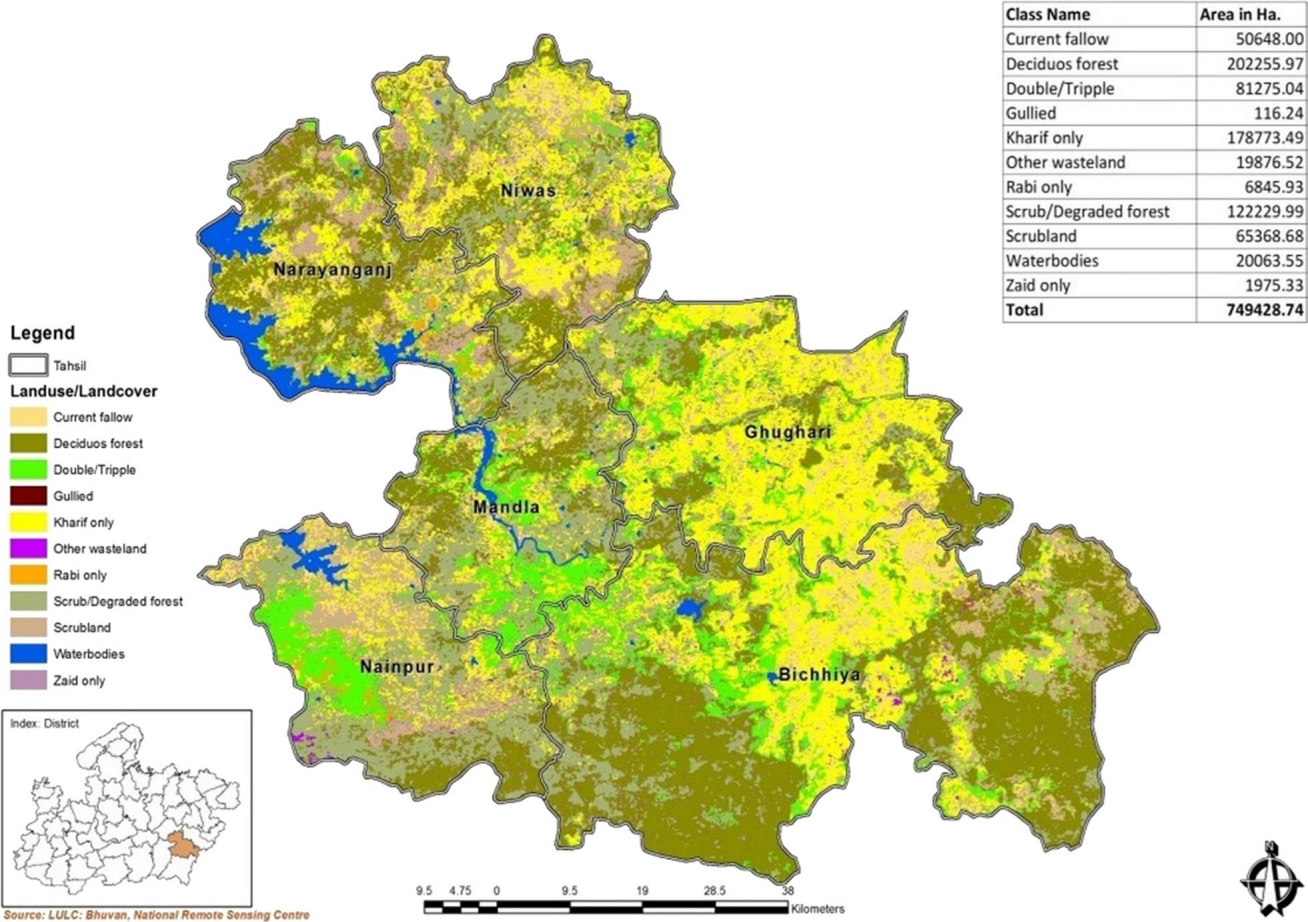
Landuse/landcover in Mandla district 2013–2014

Out of the total area of 8771 Km^2^, the district is covered by deciduous forest in over 2022 Km^2^. Major part of the forest lies in the highest malaria endemic blocks of Mawai and Bicchiya. The district also hosts a part of the National Kanha Tiger Reserve—which is located in the Bicchiya block and extends into the adjoining district of Balaghat in the south. The district has forest areas which are shown in Fig. 1 (Source—Ecological profile of Mandla by Foundation for Ecological Security) [8].

Mandla contributes to the sub-tropical climate of India and receives a moderate amount of rainfall every year. The monsoons start in the beginning of June, gains peak in July and August and phases out by September. These months are also marked by increased malaria transmission in the district. The rainfall starts in the month of May and goes till October with the July as peak month. (Source—Indian Meteorological Department, New Delhi) [9].

**Fig. 2:**
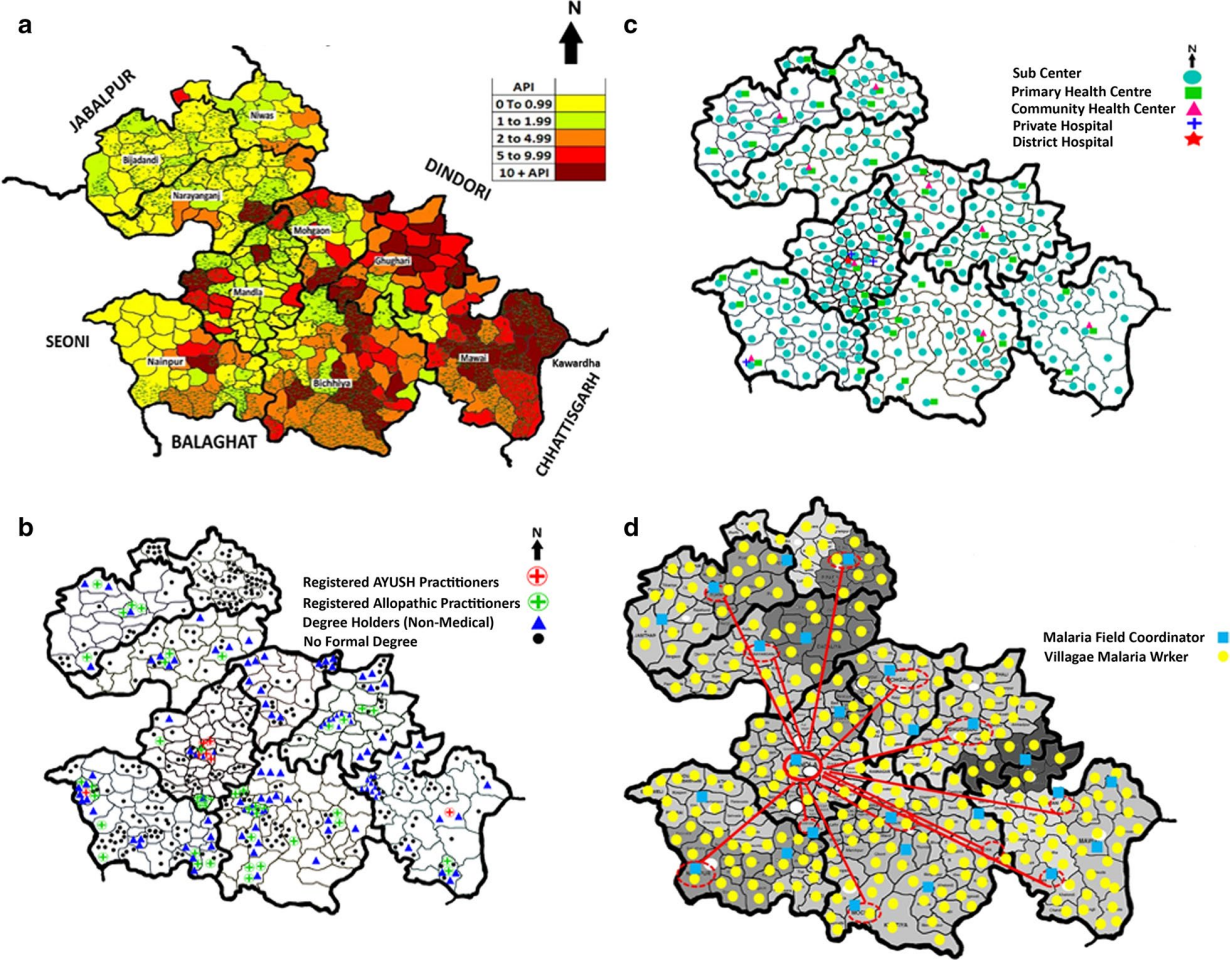
**a** Annual Parasitic Incidence map based on Mandla district epidemiological data 2015. **b** Locations of various types of private practitioners in Mandla district. **c** Location of government and private health facilities in Mandla district. **d** Reporting of hardcopy forms and samples by MEDP in Mandla district

Mandla reported 3901 malaria cases in the year 2015, and this data was used as baseline for the development of MEDP operational plan (Fig. 2a). Mandla is bordered by district Balaghat on the south which contributed 1093 cases in from April 2015 to March 2016 [10], by the state of Chattisgarh on south-east border which contributed 30,113 cases in the same period [11], by district Dindori on the east contributing 399 cases in the same period [12], by district Seoni on the west contributing 328 cases in the same period [13] and by district Jabalpur in the north contributing 127 cases in the same period [14]. The district is surrounded by malaria-endemic districts and states with high risk of inter-border transmission of malaria, thus providing opportunities for introducing malaria into Mandla district.

Apart from malaria in year 2015–2016, Mandla reported 6,090 cases of dehydration and diarrhea, 80 cases admitted with respiratory infections. These are cases reported by the government health facilities and do not account for the cases reported in the private facilities. There were 573 reported deaths due to communicable diseases, such as maternal & perinatal diseases including diarrhoea, tuberculosis, respiratory (excluding TB), malaria, other fever related and HIV/AIDS. There were 481 deaths reported due to non-communicable diseases, such as hypertension/heart disease, stroke and neurological disorders. A 110 deaths from injuries such as trauma, accidents, burns, suicide and animal bites were also reported. A total of 607 deaths also included other acute and chronic illnesses [15].

The population of Mandla uses services of private practitioners and facilities to a large extent, and many of them do not use tested and approved medical products for treatment or prevention of malaria. It was found that while there are only 7 registered allopathic practitioners in the district, there are 46 registered AYUSH (Ayurveda, Unani, Siddha & Homeopathic) practitioners, and 366 unqualified practitioners of various disciplines of medicine. Interestingly, the blocks with the highest malaria burden have the largest number of unqualified practitioners. The distribution of these practitioners and health facilities can be seen in Fig. 2b.

Mandla district has 314 functioning sub-centres (SC), 31 Primary Health Centres (PHC), 11 Community Health Centres (CHC), 1 Sub-district hospital (SHC) and 1 District hospital making a total of 358 functioning government health facilities [16] (Fig. 2c).

### Roles and responsibilities of partners

A unique aspect of this project is that it was undertaken in a public–private-partnership mode, with MEDP being responsible for the human resources, communication, management and conduct of the project. The FDEC-India has a Board of Directors and Advisory Board Members. The project follows the guidance of the Malaria Elimination Advisory Group (MEAG) that consists of experienced professionals in the field of malaria from project management (Table 1). The roles and responsibilities of the PPP stakeholders are:

A. Indian Council of Medical Research (ICMR)—National Institute of Research in Tribal Health (NIRTH). The NIRTH provides: (1) office and store space for the FDEC staff; (2) technical support for diagnosis, epidemiology, entomology, and statistical; (3) engage with NVBDCP to make sure the insecticide used in LLIN and IRS is not the same; (4) participate and coordinate with the state of Madhya Pradesh for conducting susceptibility and bioassay tests used for IRS; (5) training of FDEC staff on surveillance and case management, entomological investigations, IEC/BCC, programme guidelines; and (6) provide technical support in planning and execution of surveys, diagnostic support including microscopy and molecular testing, data collection, validation and analysis.
B. Government of Madhya Pradesh (GoMP): The GoMP provides Indoor Residual Spray of houses and/or LLINs as per the NVBDCP guideline, both these activities are monitored by FDEC India. They also provide (1) Rapid Diagnostic Kits to the project based on the actual number of fever cases detected; (2) support of ASHAs, MPWs, ANMs, and block and district level officers; (3) provide office at district-level; (4) support under the chairmanship of Collector Mandla for the project through participation of officials of departments of forest, agriculture, tribal affairs, police, education, municipalities, panchayats and horticulture. The GoMP also facilitates involvement of high school students as malaria leaders for information, education, and communication at village-level and approvals for trainings, data collection and issue letters to concerned officials for extending required cooperation.
C. Foundation for Disease Elimination and Control of India (FDEC India): The FDEC has constituted the Malaria Elimination Advisory Group (MEAG) consisting of experts in management, disease elimination and public health. The FDEC procures medicines, rapid diagnostic tests, and other equipment as needed for the project. It has the responsibility for providing supervision at operational and management levels, including hiring and removing of field staff.

**Table 1:** Malaria Elimination Advisory Group (MEAG) members

S. No.	Member
1	Director, National Vector Borne Disease Control Programme (NVBDCP), New Delhi
2	Head—Epidemiology and Communicable Diseases (ECD), ICMR, New Delhi
3	Director—ICMR National Institute of Malaria Research (NIMR), New Delhi
4	Director—ICMR National Institute for Research in Tribal Health (NIRTH), Jabalpur
5	Deputy Director (Health)—Directorate of Health Services, Government of Madhya Pradesh
6	Dr. P L Joshi, former Director—National Vector Borne Disease Control Programme, New Delhi
7	Dr. Altaf Lal, Senior Advisor—Sun Pharmaceuticals, Board member—APLMA, RBM and FDEC India, Acting Project Director—MEDP
8	Dr. Nilima Kshirsagar, National Chair of Clinical Pharmacology—ICMR, Board member—FDEC India
9	Dr. A P Dash,Vice Chancellor—Central University of Tamil Nadu, Past Director—ICMR NIRTH, Board member—FDEC India
10	Dr. A H Khan, Senior Vice President—Sun Pharmaceuticals, Board member—FDEC India
11	Dr. T Jacob John, Professor Department of PSM CMC Vellore, Advisory Board member—FDEC India
12	Dr. Pawan Ganghoria, Head, Department of Pediatrics, NSCB Medical College, Jabalpur
13	Dr. Y K Gupta, Ex Dean, All India Institute of Medical Sciences (AIIMS), New Delhi
14	Dr. Madan M Pradhan, Government of Odisha, India
15	Dr. Rajiv Tandon, RTI International
16	Chief Medical and Health Officer, Mandla, Madhya Pradesh, India
17	District Malaria Officer, Mandla, Madhya Pradesh, India
18	Dr. Shailendra Gupta, Representative of Indian Medical Association, Mandla, Madhya Pradesh, India

### Timeline

The timeline for initiation of the project is as follows. On April 25th, 2016 a statement of intent on collaboration between Indian Council for Medical Research (ICMR) and Sun Pharmaceutical Industries ltd (SPIL) was signed towards identifying areas of mutual interest in disease surveillance and disease elimination that are relevant to India. Following signing of this agreement, the parties decided to work on a Malaria Elimination Demonstration Project (MEDP), with a goal to strengthen the existing surveillance system in the Mandla district, enhance the capacity of the ASHAs and other frontline workers of MEDP through systematic capacity building and using uniform training protocols and moving towards elimination with operational research support mainly on the much needed entomological parameters, IEC/BCC and to move ahead with an all-encompassing knowledge on the essential research areas essential for malaria elimination in the district in particular and the national programme in general.

On September 21st, 2016—Sun Pharmaceuticals established Foundation for Disease Elimination and Control of India, as a Section 8 not-for–profit Company. The Malaria Elimination Demonstration Project (MEDP) was approved by FDEC as the first project. On November 16th, 2016, a three-party agreement between Government of Madhya Pradesh, Indian Council for Medical Research and Sun Pharmaceuticals was signed to conduct a malaria elimination demonstration project in 1233 villages of district Mandla.

**Fig 3:**
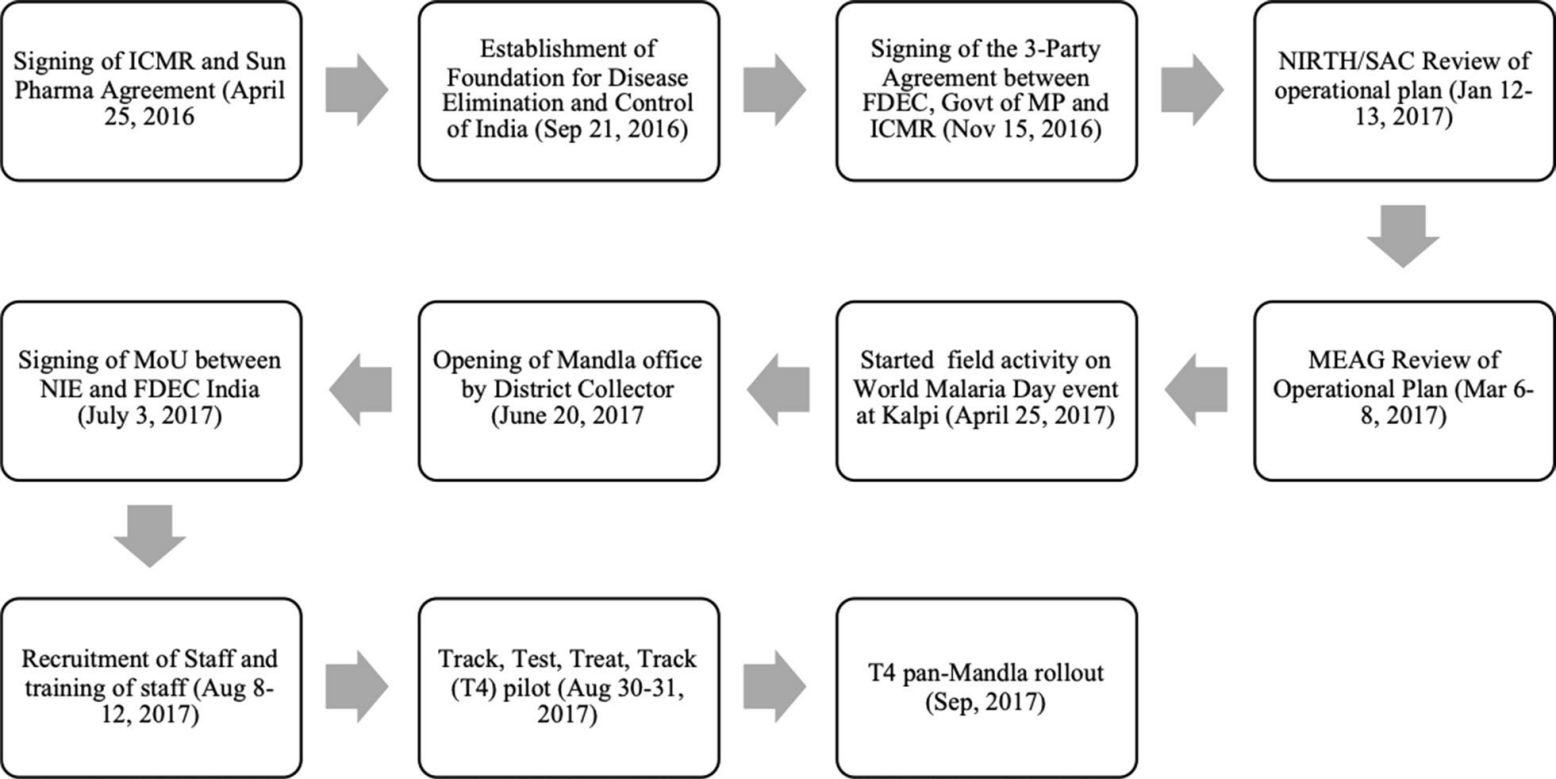
Flowchart showing timeline of project inception and implementation

The operational plan for the Malaria Elimination Demonstration Project was reviewed by Scientific Advisory Committee of Indian Council for Medical Research—National Institute of Research in Tribal Health, Jabalpur. After the review at NIRTH, the operational plan was reviewed by the Malaria Elimination Advisory Group (MEAG). Subsequently, the project was approved by the Institutional Ethical Clearance (IEC) Committee of ICMR NIRTH (reference no. 201701/10). Finally, on August 30 2017, the project was initiated with 2-day pilot launch and then district wide roll out. The project involves tracking, testing, treating, and tracking (T4) using approved diagnostic tests and drugs. This activity included hand-holding exercises of workers for all aspects of their work on the project (Fig. 3).

The goal of the project is to demonstrate elimination of malaria and prevention of re-establishment of malaria in district Mandla is feasible using robust surveillance, case management and vector control strategies. To interrupt the local transmission of malaria in Mandla district (which means there is no locally transmitted malaria), project uses evidence-based proven strategies of case management (rapid diagnosis and prompt treatment of malaria case), Integrated Vector Management (indoor residual spray, minor engineering, long-lasting insecticide treated nets), robust surveillance system and appropriate Information Education Communication (IEC) and Behaviour Change Communication (BCC) strategy.

**Fig. 4:**
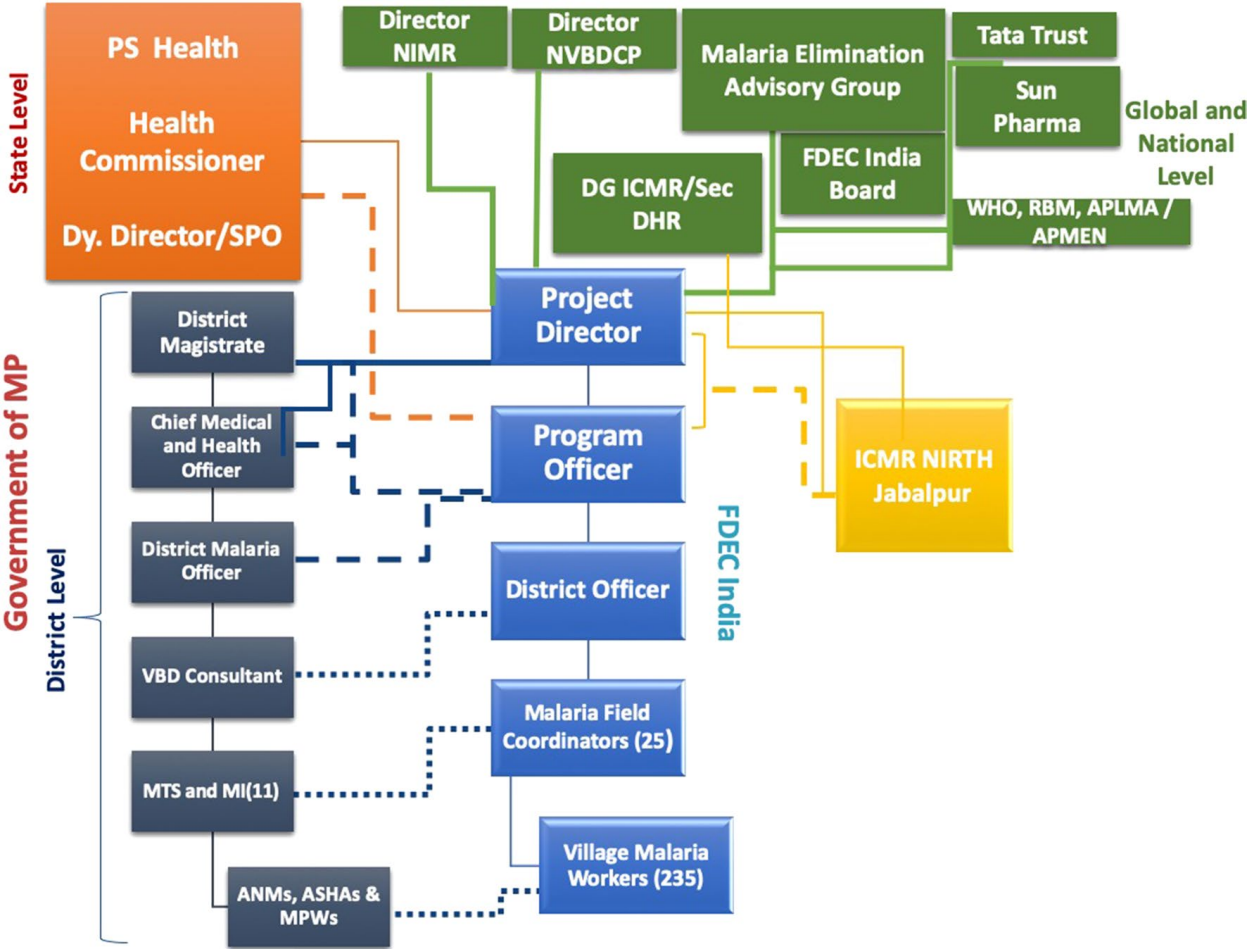
Communication between various stakeholder of Malaria Elimination Demonstration Project

Following strategies were used for achieving the goal of the study: (1) Follow guidance from NVBDCP; (2) Work closely with the State Programme Officer (SPO), Chief Medical and Health Officer (CMHO), District Malaria Officer (DMO), Block Medical Officers (BMO), Vector Borne Disease (VBD) Consultant; (3) Inform stakeholders and collaborators in real-time; (4) Work with team-work approach with local, state and national stakeholders; (5) Identify collateral benefits on other vector-borne disease, such as dengue; (6) Demonstrate benefits of intersectoral approach; and (7) Finally, develop a model for malaria elimination using lessons learned from the Mandla project. The project consists of a Project Director and team for active implementation of project at ground level. The communication between the three parties of the project at district, state and national level is presented in Fig. 4.

### Robust surveillance and case management

The project uses Track Test, Treat and Track (T4) strategy for malaria surveillance, which is modified from the T3, Track, Test and Treat strategy of the WHO [17]. The project has a staff of trained 235 Village Malaria Workers (VMWs) working pan-district. Each VMW has responsibility of door-to-door active surveillance in 6–8 villages. Each household is visited within an interval of 7–14 days depending upon the size and population of the working area. These VMWs are supervised by 25 Malaria Field Coordinators (MFCs), who are posted at the cluster-level. The Government of Madhya Pradesh has 2,184 health staff in the district that work on malaria elimination full time or part time. In the state system, the ANM and MPW conduct active surveillance, which correspond to the MEDP’s VMWs and MFCs. While MEDP has 25 MFC positions at cluster level, the state has MTS/MI at block level. At the district level, the District Malaria Officer and Vector Borne Disease (VBD) Consultant have similar roles to that of the Programme Officer and District Officer of MEDP, respectively (Fig. 4). The MEDP’s 260 field staff has allowed us to conduct robust active surveillance and administer diagnostic tests for prompt treatment.

The VMWs and MFCs were given an exhaustive and rigorous 5-days training, which includees pre- and post-training assessment. The training is administered to candidates who have cleared interview with written test and are willing to work in the proposed project locations. The passing threshold for the training was set at 70%. Training modules are composed of information on the conduct of RDT for rapid diagnosis, use of ACT for prompt treatment, and the use of vector control strategies, such as IRS, LLINs. Each VMW follows an Advance Tour Plan (ATP). This ATP consists of a concrete roster, which is micro-planned up-to the level of time of day and the village the field staff has to accomplish. An illustration of ATP is presented in Table 2. This micro-ATP enables the project to track the VMWs at any time within a defined geographical location that is sub-division of the village, thus, reducing the dependency on mobile phone communication, which does not provide reliability owing to poor telephone signals in several areas.

**Table 2:** Schematic for planning an Advance Tour Plan (ATP)

Advance Tour Plan of VMWs and MFCs								
Day	Village name	9 AM to 11.30 AM		11.30 AM to 1.30 PM		1.30 PM to 2.30 PM	2.30 PM to 5 PM	
Monday	A	A.1	VMW #1	A.2	VMW #2	Lunch + travel buffer	A.3	VMW #3
Tuesday	B	B.1	VMW #4	B.2	VMW #4		B.3	VMW #5
Wednesday	C	C.1	VMW #6	C.2	VMW #6		C.3	IEC activity (Community)
Thursday	D	D.1	VMW #7	D.2	VMW #8		D.3	VMW #8
Friday	E	E.1	IEC activity (School)	E.2	VMW #9		E.3	VMW #10
Saturday	F	F.1	Left VMW during week	F.2	Left VMW during week		F.3	Cluster-level meet with VMWs

Each Malaria Field Coordinator (MFC) supervises 6–10 VMWs, depending on the geography and population. MFCs also follow an ATP which is changed on first of every month and is only privy to the District Officer and above officials in the project. The MFC monitors and guides VMWs under his/her supervision. The MFCs administer a 30-point monitoring checklist to the VMW during every visit and submits the report to the office for evaluation. Both MFCs and VMWs are tracked by the Programme Officer and District Officer of the project up to 5 times a week during unannounced and random checks and inspections. Any deviation from the ATP has to notified in-advance and is recorded in the mobile application surveillance tool—SOCH (Solution for Community Health-workers) developed independently by the project.

The VMWs have updated census data of the entire district and visit each household mark the individuals consisting of a unique ID as: Absent, Present, House locked, Fever present. The data is live and can be updated by each VMW belonging to his/her respective villages.

**Fig. 5:**
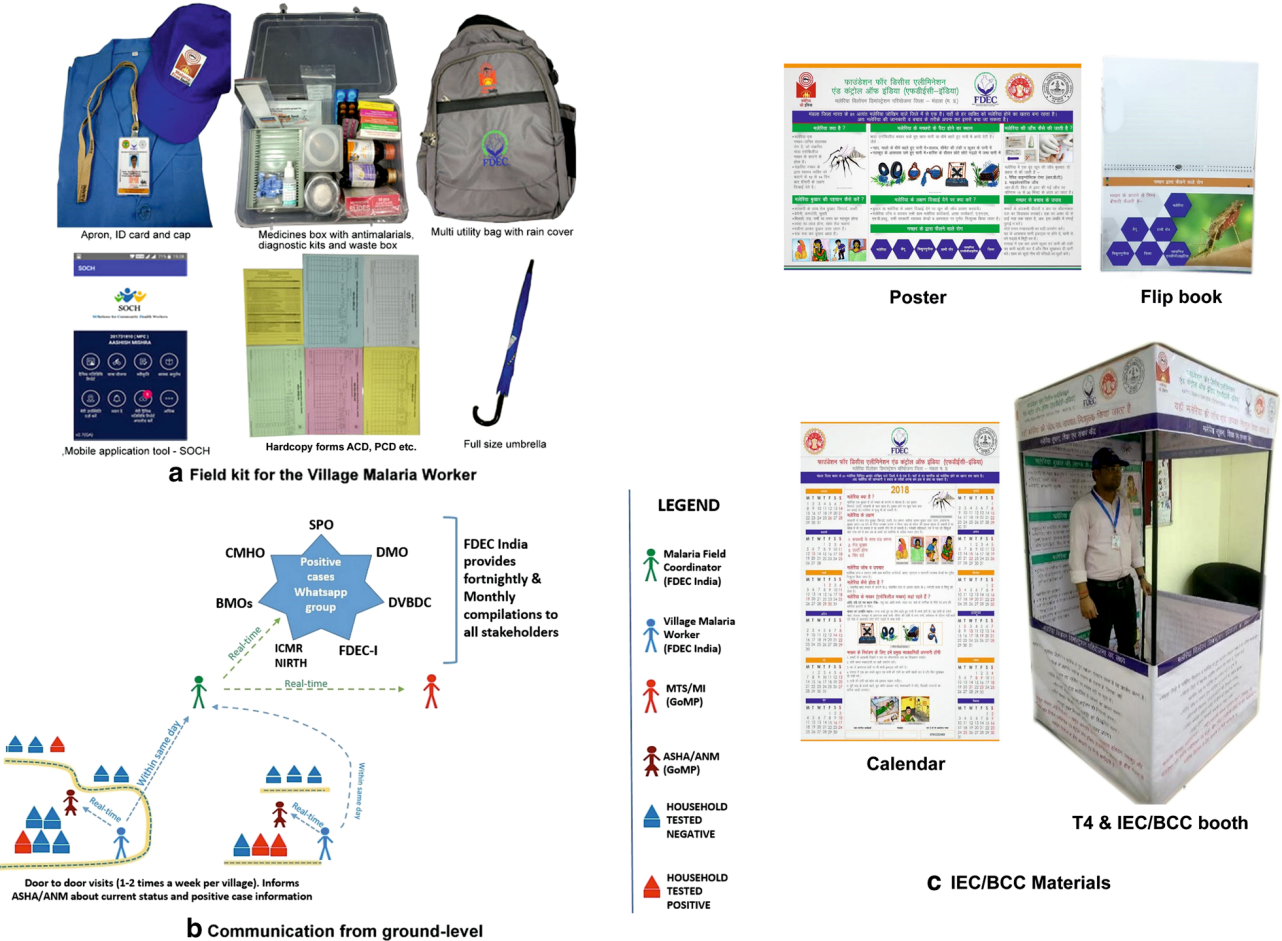
**a** Field kit of a Village Malaria Worker in MEDP Mandla. **b** Communication from ground to district level at MEDP Mandla. **c** IEC/BCC materials developed and used by MEDP Mandla

For diagnosis and treatment of malaria, the VMWs are equipped with all diagnostic and treatment modalities in their field bags (Fig. 5a). RDT is administered and if the result is negative, Paracetamol tablets for 24 h as per the age-group are dispensed by the staff and the subjects are referred to the nearest government health facility for follow-up.

For malaria positive cases, treatment is immediately dispensed as per the type of infection (*Plasmodium vivax/Plasmodium falciparum/*mixed) and age of the patient. Contact RDT is performed for all family members. Local government health functionaries are informed, family members are requested to ensure treatment compliance, local leaders are informed about the malaria case in their village and treatment follow-up is done by the VMW/MFC every alternate day. The drugs used by the project are the same as used by the state government. For Ante Natal Care (ANC) cases, the patient is referred and tracked to the nearest government health centre, where a physician is available. The project does not undertake treatment for ANC cases.

Positive case reporting is done in real-time via WhatsApp in a group containing all district-level malaria personnel for prompt notification and response. It includes Chief Medical & Health Officer, Block Medical Officers, Malaria Inspectors, Malaria Technical Supervisors, Malaria Field Coordinators, MEDP district staff. A schematic of this reporting is described in Fig. 5b. Regular reports of household visits and line-list of febrile cases are notified to the district office via SOCH mobile app. The application works in offline mode and can be synchronized with the server via internet at the end of the day.

The project also collects blood slides and filter paper samples for every 10th febrile case for microscopy and PCR based assays, respectively. These samples are transported to the district office for fixing and sent to ICMR NIRTH for further investigations. A schematic of reporting of monitoring checklists, samples and any other material can be visualized from Fig. 2d.

### Vector control

The MEDP staff monitors Indoor Residual Spray (IRS) and post-distribution usage of Long-Lasting Insecticidal Nets (LLINs) undertaken by the Government of Madhya Pradesh. Indoor Residual Sprays are done twice in a year. In the year 2017, IRS was done in sub-centers with API 1 to 4.99, MEDP staff monitored the process using an exhaustive IRS monitoring checklist tool and submitted the same to the state with recommendations. In 2018, the state sought help from MEDP to provide additional supervision and support from MFCs and VMWs in the district, same was done and the process was monitored.

LLINs were distributed in 2017 by the state in areas with API > 5, these LLINs were monitored by MEDP staff for post-distribution usage. The findings with recommendations were communicated to the state. In the year 2019, the state planned to distribute more LLINs in sub center areas with API > 2 and invited MEDP to participate in the same. Following the distribution in 2019, dependency on IRS has been decreased and more awareness activities were performed in LLIN areas to increase compliance and modify behaviors. Routine entomological investigations were also performed on a quarterly basis. These investigations help us monitoring of the quality of IRS and study the vector behaviour and biology.

### IEC/BCC activities

The project places significant emphasis on the Information Education Communication (IEC) and Behaviour Change Communication (BCC) at community level (Fig. 5c). MEDP has developed IEC/BCC material consisting of calendars, flipbooks, job-aids, posters, and booths, using feedback from the community. Currently, IEC/BCC activities are being performed in middle schools, community markets (*haat bazaars)* and as part of regular door-to-door fever surveillance. The material developed has not been copyrighted by the project, and the stakeholders are encouraged to use the material as per their requirements. The material has been provided to several state government’s malaria programmes.

### Capacity building and continuous monitoring and learning

The project has worked on capacity building of the VMWs/MFCs along with Accredited Social Health Activists (ASHAs), Auxiliary Nurse Midwifery (ANMs) and Multi-Purpose Workers (MPWs) of the state. An exhaustive needs-assessment on malaria knowledge and practices of ASHAs of the district was undertaken by the project and findings along with the recommendations were shared with the state. Following which, MEDP was invited to participate in the trainings of 1000 + ASHAs of the district. The project also follows a robust review mechanism of various project components (Table 3).

**Table 3:** Tools and methods used for monitoring and continuous learning of various project components

S.No.	Project component/process	Frequency	Mode	Follow-up/action taken/review
1	Overall project progress—Malaria Elimination Advisory Group (MEAG) meeting	Annual	In-person	In the next meeting
2	Monthly project progress reports—includes surveillance data and major highlights of the month	Monthly	Electronic	In the subsequent month
3	Weekly project progress report—includes work-done, work-planned, open action items and detailed field visit reports	Weekly	Electronic report followed by in-person conference every Monday	Immediate
4	MFC-level monthly meeting	Monthly	In-person discussion	Immediate
5	VMWs meeting at cluster-level	Fortnightly	In-person discussion	Immediate
6	30-point monitoring checklist	Monthly compilation	Hardcopies—digitized within same month	In subsequent months
7	Findings during field visits	Weekly	Electronic reports and in-person discussions	Immediate
8	Work report of each MFC and VMW with highlights	Daily	Electronic on SOCH app and WhatsApp	Immediate
9	Monitoring and feedback of work-products through mobile app and WhatsApp	24 × 7	Verbal & electronic through WhatsApp	Immediate

### Integrated malaria and COVID-19 surveillance

In March 2020, a pan-India lockdown was announced by the Government of India to control the transmission of COVID-19 in the country. MEDP was requested by the Government of MP to undertake the tracking, screening, and isolation of patients with Severe Acute Respiratory Illness (SARI) and Influenza-like Illness (ILI) in the entire district. The Advance Tour Plans of the field staff were revised to cater the new responsibilities without disrupting the existing efforts in malaria elimination. The VMWs and MFCs of MEDP now track the suspected COVID-19 patients using their door-to-door surveillance strategies and migration databases along with regular screening of fever cases for malaria. The staff has been trained in infection-control guidelines specific to COVID-19 and have been provided with Personal Protection Equipment (PPE), such as masks, gloves, and hand-sanitizers. The initial screening by MEDP populates a list of suspected COVID-19 patients and are shared in real-time with the state authorities for further action.

## Results

The project started its field operations from September 2017. The data from June 2017 to August 2017 has been provided by the District Malaria Office. During this period, a total of 357,143 febrile cases were screened using RDTs, out of which, 673 (0.19%) tested positive for malaria, which were promptly treated. during the first year (June 2017 to May 2018), the malaria positivity rate of indigenous cases was 0.30 (377/123,641), while in year 2 it was 0.16 (169/103,143), and in year 3 it was 0.01 (127/130,359) (*p* < 0.001). This 91% reduction was achieved through conventional vector control and case management strategies, with stringent management and operational controls at staff and supply-chain levels and through weekly in-depth reviews.

In 2016, when the operational plan was being developed, the data from Government of MP revealed that a total of 77 sub-centres were free from malaria. Out of the total 297 sub-centres, in 2017, 143 sub-centres were free of malaria, which increased to 198 in 2018, and 211 in 2019. Out of these sub-centres, few sub-centres were identified with greater than five API (malaria hotspots). These hotspots were targeted using different vector control interventions over the course of 24 months with continuous active surveillance and case management. These eight hotspots in 2016 reduced to three in 2017, further to two in 2018, and dropped to zero in 2019.

## Discussion

The project has developed a robust surveillance system that utilizes Track, Test, Treat, and Track (T4) for malaria elimination at district-level [18]. This system together with monitoring and evaluation and operational controls is providing valuable knowledge that could be used to advance elimination goals at state and central level. There is need for effective coverage of core malaria interventions for all populations at risk and using high-quality surveillance data for decision-making. The project has completed 33 months of its field operations and the results are presented in the series of eleven MEDP companion papers that present findings on gaps in human resources, development and usage of mobile app for disease surveillance, workforce management and supply chain management, proper utilization of vector control tools (IRS and LLINs), development of context specific IEC campaigns for behaviour change, and reduction of malaria cases over the past 33 months.

In the current pandemic, MEDP is managing the responsibilities of COVID-19 and malaria surveillance in a planned and systematic manner. The existing surveillance strategies have seamlessly integrated with tracking of suspected COVID-19 patients in the district without compromising the efforts for malaria elimination. A detailed model for malaria elimination based on the learnings of MEDP has also been prepared to help the national malaria elimination efforts.

## Conclusion

This paper outlines the study design and the operational plan for malaria elimination in a high-burden district of Central India, which presented difficulties of hard to reach areas, forest malaria, and complex epidemiology of urban and rural malaria. The plan has been developed by subject matter experts from the fields of malaria/disease elimination, policy, public health, advocacy, research, as a Malaria Elimination Advisory Group. The lessons learned from MEDP could be used for malaria elimination efforts in rest of the country and other parts of South Asia with comparable demography and epidemiology. The results provide optimism and belief that malaria elimination in India is feasible in less than a decade, even in challenging situations such as the on-going COVID-19 pandemic, when appropriate resources are deployed with proper accountability, monitoring, management controls and independent programme reviews.

## Data Availability

We have reported all the findings in this manuscript. The hardcopy data is stored at MEDP Office in Mandla, Madhya Pradesh and Indian Council of Medical Research- National Institute of Research in Tribal Health (ICMR-NIRTH), Jabalpur, Madhya Pradesh. Softcopy data is available on the project server of MEDP hosted by Microsoft Azure. If anyone wants to review or use the data, they should contact: Dr. Altaf A. Lal, Project Director—Malaria Elimination Demonstration Project, Mandla Foundation for Disease Elimination and Control of India, Mumbai, India 482003. E mail: altaf.lal@sunpharma.com.

## Abbreviations

ANC: Ante Natal Care
ANMs: Auxiliary Nurse Midwife
API: Annual Parasite Incidence
ASHA: Accredited Social Health Activist
ATP: Advance Tour Plan
BCC: Behavior Change Communication
BMO: Block Medical Officer
CHC: Community Health Center
CMHO: Chief Medical and Health Officer
COVID-19: Coronavirus Disease 2019
DMO: District Malaria Officer
EAS: East Asia Summit
FDEC: Foundation for Disease Elimination and Control
GoMP: Government of Madhya Pradesh
ICMR: Indian Council of Medical Research
IEC: Information Education Communication
IRS: Indoor Residual Spray
LLINs: Long-Lasting Insecticidal Nets
MEAG: Malaria Elimination Advisory Group
MEDP: Malaria Elimination Demonstration Project
MFC: Malaria Field Coordinator
MPW: Multi-Purpose Worker
NFME: National Framework for Malaria Elimination
NIRTH: National Institute of Research in Tribal Health
NMCP: National Malaria Control Programme
NSP: National Strategic Plan
PHC: Primary Health Centers
PPP: Public–Private-Partnership; SC: Sub-Centre
